# Preoperative flexion contracture is a predisposing factor for cartilage degeneration at the patellofemoral joint after open wedge high tibial osteotomy

**DOI:** 10.1186/s43019-020-00063-2

**Published:** 2020-10-13

**Authors:** Shuhei Otsuki, Kuniaki Ikeda, Hitoshi Wakama, Nobuhiro Okuno, Yoshinori Okamoto, Tomohiro Okayoshi, Yuki Miyamoto, Masashi Neo

**Affiliations:** Department of Orthopedic Surgery, Osaka Medical College, 2-7 Daigakumachi Takatsuki, Osaka, 569-8686 Japan

**Keywords:** Knee, Open wedge high tibial osteotomy, Range of motion, Cartilage degeneration, Patellofemoral joint, Clinical outcome

## Abstract

**Purpose:**

The purpose of the study was to determine the effect of cartilage degeneration at the patellofemoral joint on clinical outcomes after open wedge high tibial osteotomy and to investigate the predisposing factors for progressive patellofemoral cartilage degeneration.

**Methods:**

Seventy-two knees were evaluated on second-look arthroscopy in patients who opted for plate and screw removal at an average of 20.1 months after osteotomy. Cartilage degeneration at the patellofemoral joint was evaluated using the International Cartilage Repair Society grading system, with cases divided into progression and nonprogression groups. Radiographic parameters of the patellofemoral anatomy, knee range of motion, and clinical outcomes were evaluated from the preoperative baseline to the final follow up, on average 50 months after osteotomy. A contracture > 5° was considered a flexion contracture.

**Results:**

Cartilage degeneration progressed in 31 knees, and preoperative knee flexion contracture was significantly associated with progressive degeneration (*P* < 0.01). The Lysholm and Kujala scores were significantly lower in the progression group (87.9 and 85.3, respectively) than in the nonprogression group (91.6 and 93.6, respectively) (*P* < 0.05). The odds ratio of the flexion contracture resulting in progression of patellofemoral cartilage degeneration was 4.63 (95% confidence interval, 1.77–12.1). No association was detected between progressive degeneration and age, sex, body mass index, Kellgren-Lawrence grade, or radiographic parameters.

**Conclusions:**

Flexion contracture may be associated with progression of cartilage degeneration at the patellofemoral joint and may negatively affect the clinical outcomes after open wedge, high tibial osteotomy.

## Introduction

Open wedge high tibial osteotomy (OWHTO) is a successful treatment option for osteoarthritis (OA) in the medial compartment of the knee [1, 2]. Despite the favorable clinical outcomes of OWHTO, complications have been reported, such as loss of correction, nonunion, plate irritation, device failures, hinge fracture, and patella baja [3–5]. Particularly, the progression of postoperative patellofemoral cartilage degeneration is an important clinical issue to consider [5–7]. The degree of correction angle [6], overcorrection [7], and sagittal osteotomy inclination [8] have been recognized as risk factors for patellofemoral OA.

Knee range of motion (ROM) is an essential factor for good clinical outcomes after high tibial osteotomy (HTO) [9]. A significant decrease in ROM has been reported in patients with coexisting patellofemoral and femorotibial OA, compared with those with isolated femorotibial OA [10]. The increase in contact pressure at the patellofemoral joint due to flexion might accelerate cartilage degeneration [11], with a specific increase in patellofemoral joint contact pressure within the first 20° of knee flexion [12]. As such, the presence of a knee flexion contracture after OWHTO may critically increase the risk of patellofemoral cartilage degeneration after surgery. The aim of this study was to determine the effect of cartilage degeneration at the patellofemoral joint on clinical outcomes after OWHTO and to investigate the predisposing factors for progressive patellofemoral cartilage degeneration.

## Materials and methods

### Ethics statement

Ethical approval for this study was obtained from the institutional review board of our institution, and written informed consent was provided by all patients.

### Study group

From November 2015 until July 2017, 108 patients (112 knees) were treated using biplanar OWHTO. The surgical indications for OWHTO were medial compartment knee OA or spontaneous osteonecrosis of the knee, bony correction angle requirement < 15° based on the preoperative planning, body mass index (BMI) < 35 kg/m^2^, absence of or well-controlled diabetes mellitus, preoperative knee flexion contracture ≤ 10°, and a minimum of 120° of knee ROM. The contraindications were symptomatic lateral compartment knee OA, symptomatic patellofemoral OA, flexion contracture > 15°, and anterior or posterior cruciate ligament deficiency. There was no age restriction. The exclusion criterion was < 1 year of second-look arthroscopy at the time of plate removal. Of these, the 72 patients who opted to undergo removal of the plate and screw because of discomfort or pain, and agreed to a concomitant arthroscopic evaluation were enrolled in this study. The mean age of the patients included in our study was 62.7 ± 11.1 (range 44–78) years, with a mean BMI of 24.6 ± 2.8 (range 20.8–34.9) kg/m^2^. Tibiofemoral OA was evaluated using the Kellgren-Lawrence (K/L) grade with the distribution of K/L grades as follows: grade I, 1 patient; grade II, 19 patients; grade III, 35 patients; and grade IV, 17 patients. The average time from OWHTO to second-look arthroscopy was 20.1 ± 4.1 (range 12–26) months.

### Surgical procedure and assessment

The aim of OWHTO is to correct the mechanical axis of the knee joint [13]. Preoperative planning for HTO first considers the intended postoperative mechanical axis, which passes through the lateral tibial eminence on the coronal view. This axis was determined using the digital planning software (TraumaCaD; Brainlab, Feldkirchen, Germany) in the Picture Archiving and Communication System. Arthroscopy was routinely performed prior to surgery to evaluate the degree of cartilage degeneration, quantified using the International Cartilage Repair Society (ICRS) grading system [14]. The surgical procedure used for OWHTO has previously been described [15]. Briefly, the medial proximal tibia was exposed using a J-shaped incision, and the superficial medial collateral ligament and the pes anserinus were released. Two Kirschner wires (K-wires) were inserted into the proximal tibiofibular joint 35–40 mm inferior to the knee joint line, fixed with a locking plate (TomoFix; DePuy Synthes, Solothurn, Switzerland, or Tris Medial HTO Plate System; Olympus, Tokyo, Japan) and used as a guide. The gap created by the osteotomy was filled with β-tricalcium phosphate (Olympus). All surgery was performed by the same surgeon (senior author). Rehabilitation was initiated on postoperative day 2, with full weight-bearing permitted as tolerated by the patient.

### Diagnostic and radiographic measurements

Preoperative and postoperative knee alignments were compared using the hip-knee-ankle angle (HKA), defined as the angle between the mechanical axes of the femur and the tibia, with the percent change in the alignment of the mechanical axis (%MA) quantified for analysis. Specifically, to quantify the MA, a line was drawn from the center of the femoral head to the center of the ankle joint, with the point of intersection of this line with the tibial plateau expressed as a percentage of the tibial width. The medial proximal tibial angle (MPTA) and the posterior tibial slope were also calculated [16]. The position of the patella was evaluated using the Caton-Deschamps index and the tibial tuberosity to the trochlear groove (TT-TG) distance [17] and the patellar tilt was measured on computed tomography (CT) images [18]. The Caton-Deschamps index was determined by measuring the distance from the distal end of the patellar joint surface to the anterior tip of the tibial tuberosity and dividing this value by the length of the patellar joint surface. Measurements were obtained on lateral view radiographs, with the knee in 30° of flexion [17]. To evaluate the possible association between limited knee ROM and patellofemoral OA, the active flexion and extension ROMs of the affected knee joint were measured by two orthopedic surgeons using standard goniometric procedures. The average ROMs measured by the two orthopedic surgeons were analyzed. A contracture > 5° was considered a flexion contracture. Clinical outcomes were evaluated using the Lysholm and Kujala scores. All outcome parameters were evaluated before surgery and at the final follow up, 50.3 ± 7.2 (range 40–62) months after surgery. All measurements were independently performed by two orthopedic surgeons, and these parameters were compared between those with (group P) and without progression of cartilage degeneration (group N) to determine the predisposing factor for progression of patellofemoral cartilage degeneration.

### Statistical analysis

A power analysis was performed, which indicated that 21 cases were required in each group to yield power of 0.8, a significance level of 0.05, and an effect size of 0.8. The power of the association between patellofemoral OA and ROM was > 0.9, as calculated using G*Power version 3.1.9.2 (Heinrich Heine University Düsseldorf, Düsseldorf, Germany) [19]. Progression of cartilage degeneration after OWHTO was evaluated using the Wilcoxon *t* test because the data were not normally distributed according to the normality test. Factors related to the progression of patellofemoral OA were evaluated using the Mann-Whitney *U* test. The paired *t* test was used to compare the preoperative and postoperative radiographic results. The odds ratio of a loss of full knee extension on the progression of patellofemoral joint degeneration was calculated. These analyses were performed using JMP Pro version 13 (SAS Institute Inc., Cary, NC, USA), with *P* < 0.05 considered statistically significant. Interobserver and intraobserver reliability for the ICRS cartilage score (quantified during arthroscopy) and ROM was evaluated using SPSS version 21.0 (IBM Corp., Armonk, NY, USA), with *P* < 0.05 considered statistically significant. The intraobserver and interobserver reliability for the ICRS cartilage grading using arthroscopy was 0.881 (95% confidence interval (CI) 0.776–0.937) and 0.842 (95% CI 0.720–0.913), respectively, and 0.981 (95% CI 0.858–0.983) and 0.903 (95% CI 0.504–0.983), respectively, for ROM. Therefore, all radiographic parameters were measured with high reliability and reproducibly [15].

## Results

**Fig. 1:**
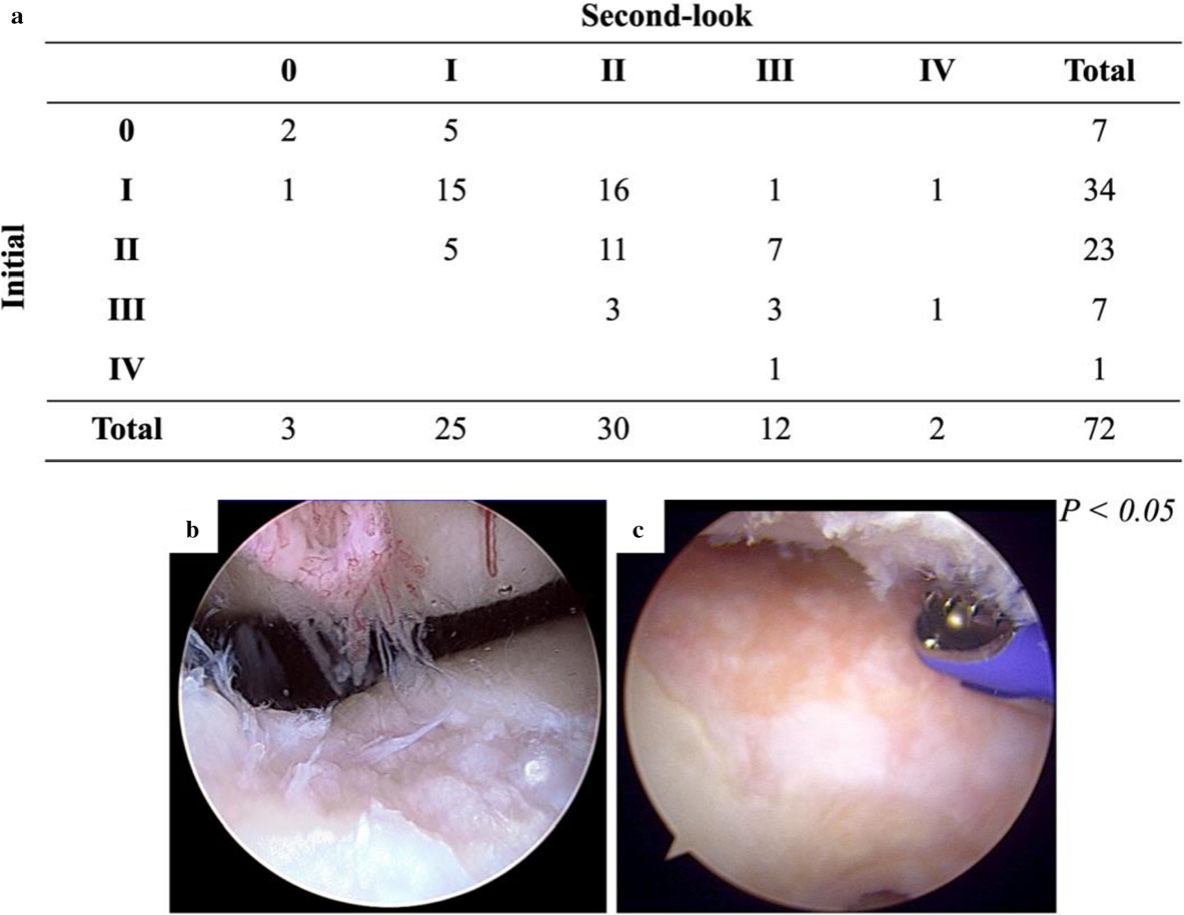
Patellofemoral cartilage degeneration. **a** Comparison of cartilage degeneration between the initial and second look arthroscopy. **b**, **c** Representative case of progressive cartilage deterioration after open wedge, high tibial osteotomy (OWHTO) in a 58-year-old man; femoral-trochlear cartilage progressed from International Cartilage Repair Society (ICRS) grade II before surgery to ICRS grade IV at the final follow up

Cartilage degeneration at the patellofemoral joint progressed in 31 of 72 patients who underwent second-look arthroscopy (Fig. 1a, *P* < 0.05). A representative case of progressive patellofemoral cartilage deterioration after OWHTO is shown in Fig. 1b and c, with a preoperative cartilage ICRS degeneration grade II (Fig. 1b) progressing to a grade IV after OWHTO (Fig. 1c). The patients were divided into groups P (*n* = 31) and N (*n* = 41). The demographic data (Table 1) and radiographic parameters (Table 2) were not significantly different between the two groups. Group P had significantly greater preoperative knee flexion contracture than group N (Table 3, *P* < 0.01). However, the postoperative ROM was not significantly different between the two groups. With regard to clinical outcomes, the Lysholm and Kujala scores were 87.9 and 85.3, respectively, in group P, which were significantly lower than those in group N (91.6 and 93.6, respectively) (Table 3, *P* < 0.05). The odds ratio of a loss of full knee extension resulting in progression of patellofemoral cartilage degeneration was 4.63 (95% CI 1.77–12.1) (Table 4).

**Table 1:** Demographic data

	Progression of patellofemoral OA (group P: *n* = 31)	No progression (group N: *n* = 41)	*P* value
Age, years	63.9 ± 11.3	61.8 ± 11.1	0.49
Sex, male/female	12/ 19	17/ 24	0.81
BMI, kg/m^2^	25.4 ± 3.2	24.1 ± 2.5	0.11
K/L grade			
I	0	1	
II	8	11	
III	18	17	
IV	5	12	0.38
Time from OWHTO to second-look arthroscopy (months)	19.6 ± 4.4	20.4 ± 6.3	0.58

*BMI* body mass index, *K/L* Kellgren/Laurence, *OA* osteoarthritis, *OWHTO* open-wedge, high tibial osteotomy

**Table 2:** Comparison of radiographic data between the patellofemoral OA progression and non-progression group

	Preoperative		*P* value	Postoperative		*P* value
	Group P	Group N		Group P	Group N	
**Correction angle (degrees)**				9.0 ± 2.8	8.7 ± 2.1	0.80
**HKA (degrees)**	6.0 ± 4.6 varus	5.9 ± 4.9 varus	0.96	3.1 ± 2.5 valgus	2.9 ± 2.2 valgus	0.75
**Percentage MA**	21.0 ± 11.6	23.5 ± 9.9	0.38	60.1 ± 6.5	60.2 ± 5.2	0.95
**MPTA (degrees)**	82.5 ± 2.4	83.4 ± 1.5	0.10	91.7 ± 2.1	92.3 ± 1.5	0.25
**Posterior tibial slope (degrees)**	8.6 ± 3.8	8.3 ± 3.2	0.73	8.4 ± 3.6	8.9 ± 3.6	0.64
**Patellar tilt (degrees)**	9.0 ± 4.5	9.1 ± 5.8	0.90	7.9 ± 4.2	7.1 ± 5.0	0.57
**Caton-Deschamps index**	0.94 ± 0.20	0.91 ± 0.11	0.51	0.76 ± 0.21	0.78 ± 0.12	0.63
**TT-TG distance (mm)**	11.2 ± 2.9	11.5 ± 3.4	0.79	11.7 ± 3.2	11.4 ± 2.9	0.80

*O*A osteoarthritis, *HKA* hip-knee-ankle angle, *MA* mechanical axis, *MPTA* medial proximal tibial angle, *TT-TG* tibial tuberosity-trochlea groove

**Table 3:** Comparison of ROM and clinical outcome between the patellofemoral OA progression and non-progression group

	Preoperative		*P* value	Postoperative		*P* value
	Group P	Group N		Group P	Group N	
**ROM**						
Extension (degree**s**)	- 4.4 ± 4.1	- 1.3 ± 2.5	< 0.01	- 4.6 ± 4.5	- 2.7 ± 3.1	0.07
Flexion (degrees)	144.3 ± 3.8	146.9 ± 6.1	0.08	143.9 ± 3.8	145.4 ± 5.1	0.33
**Clinical outcome**						
Lysholm score (points)	56.6 ± 8.6	57.5 ± 10.7	0.74	87.9 ± 7.6	91.6 ± 5.8	< 0.05
Kujala score (points)	54.2 ± 6.6	55.5 ± 8.7	0.61	85.3 ± 7.2	93.6 ± 5.9	< 0.05

*ROM* range of motion, *OA* osteoarthritis

**Table 4:**
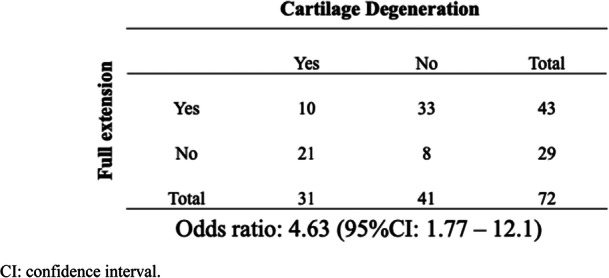
Odds ratio analysis between full extension of the knee and cartilage degeneration

## Discussion

Our results indicate that preoperative knee flexion contracture was associated with progression of cartilage degeneration at the patellofemoral joint, which affected the clinical outcomes after OWHTO. Previous studies have indicated that the severity of varus knee malalignment is associated with patellofemoral OA [18]. Therefore, correction of varus knee alignment may offer protection against progression of patellofemoral OA. Although serious complications after OWHTO are rare, cartilage degeneration at the patellofemoral joint has been recognized as one of the critical complications of OWHTO [20], Goshima et al. [21] reported progression of cartilage degeneration at the trochlear groove in 30% of patients on second-look arthroscopy at a mean follow up of 19 months after OWHTO. Our results are consistent with this finding, with progression of OA at the femoral trochlea in 43.1% of our cases. The degree of change in patellar height correlates with the magnitude of the correction angle after HTO [16]. In our case series, the correction angles after OWHTO were not severe, and thus, the correction angle was not a contributing factor of patellofemoral OA progression.

Regarding knee ROM and clinical outcomes after HTO, Berman et al. [22] reported that patients with favorable results had a preoperative range of motion of at least 90°. Naudie et al. [23] reported that a preoperative ROM < 120° was associated with early failure of HTO. However, the relationship between ROM and favorable results after HTO are still largely unknown, with no previous study having reported on the association between ROM and patellofemoral OA after OWHTO.

The patellofemoral contact pressure is significantly greater after OWHTO than that in the intact knee [24]. Moreover, Yang et al. [12] investigated the patellofemoral contact pressure in a biomechanical cadaver study and reported patellofemoral contact pressure was a 4-fold increase with the knee in 10° of flexion and a 7-fold increase at 20° of flexion compared with full extension. Therefore, the presence of a small flexion contracture (e.g., a loss of full extension) might increase contact pressure at the patellofemoral joint, leading to a possible progression to OA. Indeed, our odds ratio analysis indicated that a loss of full extension was a critical factor for progression of cartilage degeneration at the patellofemoral joint. Knee motion during daily activitiies such as climbing stairs and walking, indeces more patellofemoral contact pressure with knee flexion contracture [25]. This suggests that loss of full extension accelerates deterioration of the patellofemoral cartilage during activities of daily life after OWHTO.

Lobenhoffer and van Heerwaarden [26] identified recovery of full knee extension as an important factor for good clinical results after HTO, with 10° of flexion contracture being correctable during the OWHTO procedure. Although decreasing the posterior tibial slope may resolve a knee flexion contracture, the posterior tibial slope tended to increase after OWHTO [27], suggesting that controlling the posterior tibial slope might be difficult to achieve with OWHTO.

In terms of clinical outcomes, the Kujala score provides a more specific evaluation of patellofemoral status. In our study group, both the Lysholm and Kujala scores were significantly lower in group P than in group N at an average follow up of 4 years after the index OWHTO. These findings indicate that a knee flexion contracture after OWHTO may increase the risk of patellofemoral OA progression and thus negatively impact clinical outcomes. Therefore, based on our findings, a surgical procedure other than OWHTO should be selected for the surgical treatment of a varus knee deformity combined with a flexion contracture, such as distal tuberosity osteotomy [28] or a hybrid HTO [29], neither of which influence the patellofemoral joint.

In their assessment of prognostic factors for patellofemoral OA after OWHTO, Tanaka et al. [6] did not identify correlation between knee joint ROM and cartilage deterioration, which is different from our findings. This difference likely results from variations in patient characteristics in the two studies. Specifically, the patients in our study group were older on average, were predominantly women, and they had greater preoperative varus malalignment than the patients in the study of Tanaka et al. All three of these factors are associated with a higher likelihood of severe knee OA and a greater degree of knee flexion contracture, which would further negatively impact the homeostasis of the cartilage of the knee. We note that in our study, we only evaluated cartilage degeneration at the femoral trochlea, as a previous study indicated a similar extent of degeneration between the femoral trochlea and the patellar facets after OWHTO [6].

The limitations of our study need to be acknowledged. First, this is a retrospective study and selection bias cannot be denied, as second-look arthroscopy was performed only on patients who wanted removal of the plate. Second, although we showed the importance of flexion contracture in cartilage degeneration and clinical outcome, the standard goniometry measurement might not be sufficiently sensitive because the difference between groups was very small despite excellent interobserver and intraobserver reliabilities of ROM. Third, there was a wide variation in age across the patients included in our study. Future studies should aim to determine the effect of flexion contracture on clinical outcomes in a larger number of patients with a narrower age range.

## Conclusion

Flexion contracture may be associated with progression of degeneration of the cartilage at the patellofemoral joint and may negatively affect the clinical outcomes after open wedge, high tibial osteotomy. Although OWHTO is the first-line surgical treatment for patients with knee OA due to a varus malalignment, the indications for OWHTO should be carefully considered, even in patients with a small degree of knee flexion contracture, and full extension might be an important indication for OWHTO to avoid progression of patellofemoral OA.

## Data Availability

The datasets obtained and/or analyzed during the current study are available from the corresponding author on reasonable request.

## Abbreviations

BMI: Body mass index
CT: Computed tomography
HKA: Hip-knee-ankle angle
HTO: High tibial osteotomy
ICRS: International Cartilage Repair Society
K/L: Kellen-Lawrence
K-wire: Kirschner wire
MA: Mechanical axis
MPTA: Medial proximal tibial angle
OA: Osteoarthritis
OWHTO: Open wedge, high tibial osteotomy
PACS: Picture archiving and communications system
ROM: Range of motion
TT-TG: Tibial tuberosity to trochlear groove
